# Comprehensive characterization of lncRNA N^6^-methyladenosine modification dynamics throughout bovine skeletal muscle development

**DOI:** 10.1186/s40104-025-01164-2

**Published:** 2025-03-06

**Authors:** Cui Mao, Wei You, Yuta Yang, Haijian Cheng, Xin Hu, Xianyong Lan, Enliang Song

**Affiliations:** 1 Key Laboratory of Animal Genetics, Breeding and Reproduction of Shaanxi Province, College of Animal Science and Technology, Northwest A&F University, Yangling, 712100 China; 2 Institute of Animal Science and Veterinary Medicine, Shandong Academy of Agricultural Sciences, Jinan, 250100 China; 3 Key Laboratory of Livestock and Poultry Multi-Omics of MARA, Jinan, 250100 China

**Keywords:** Bovine, LncRNA, m^6^A methylation, Muscle development

## Abstract

**Background:**

N^6^-methyladenosine (m^6^A) methylation is a key epigenetic modification that can modulate gene expression and strongly affect mammalian developmental processes. However, the genome-wide methylation of long non-coding RNAs (lncRNAs) and its implications for the development of skeletal muscle remain poorly understood. Bovine skeletal muscle samples from five developmental stages were analyzed in this study to establish lncRNA methylome and transcriptomic maps.

**Results:**

Globally, 59.67% of lncRNAs in skeletal muscle with m^6^A modifications, and this percentage decreased progressively during development. lncRNA expression levels were positively associated with the number of m^6^A peaks, with lncRNAs possessing 3 or more peaks showing significantly higher expression levels than those with 1 or 2 peaks. Specific lncRNAs involved in skeletal muscle development were identified through two analytical approaches. The first approach employed weighted gene co-expression network analysis (WGCNA) of transcriptomic data to identify correlations between annotated lncRNAs and growth-related traits, resulting in 21 candidate hub lncRNAs. The intersection of these 21 hub lncRNAs with 151 differentially methylated lncRNAs (DM-lncRNAs) identified 10 shared candidate lncRNAs. The second approach integrated MeRIP-seq and RNA-seq data to identify 36 lncRNAs that were both differentially m^6^A modified and differentially expressed (dme-lncRNAs). GO and KEGG enrichment analyses of *cis*-target genes associated with these dme-lncRNAs identified eight candidate lncRNAs. Combining the results from the two approaches identified 16 key m^6^A-modified lncRNAs likely involved in skeletal muscle development.

**Conclusions:**

These findings highlight the regulatory and functional significance of dynamic lncRNA methylation in skeletal muscle development.

**Supplementary Information:**

The online version contains supplementary material available at 10.1186/s40104-025-01164-2.

## Background

In addition to being the most abundant tissue type in the mammalian body, skeletal muscle growth and development are the primary determinants of meat yield and meat quality for livestock [1, 2]. Studies of skeletal muscle development are thus an extremely important topic in the animal genetics and breeding field. Skeletal muscle growth involves the coordinated regulation of processes such as myoblast proliferation and differentiation across various stages of development [3]. Skeletal muscle of the trunk and limbs is of mesodermal origin, developing from dorsal portions of somites arising from mesoderm segmentation [3, 4]. This process involves the regulatory control of many transcription factors, signaling molecules, long non-coding RNAs (lncRNAs), and N^6^-methyladenosine (m^6^A) methylation residues. The influence of novel regulatory factors, including m^6^A methylation, on this process, remains partially understood, requiring further research to improve livestock meat quality through genetic improvements.

lncRNAs, a large class of RNAs over 200 nucleotides long, generally lack protein-coding potential but often exhibit poly-A tailing and splicing. They are abundant in the cytosol and nuclear compartments and have complex spatial structures and a range of functions [5, 6]. Specific lncRNAs regulate various biological processes, including development and disease progression. Altered lncRNA expression is associated with muscle-related diseases and plays a significant role in regulating muscle tissue development [7]. lncRNAs influence biological processes by competitively binding molecules, altering target protein stability, regulating translation, acting as molecular sponges, activating transcription or modifying chromosomes [7]. The competing endogenous RNA (ceRNA) mechanism is thought to be the primary pathway by which lncRNAs influence muscle development through the sequestration of miRNAs with sequence complementarity, thereby altering target mRNA expression to shape developmental processes [8–12]. *IGF2-AS* [13] and *lncMGPF* [14] are established ceRNAs that respectively bind to miR-503 and miR-135a-5p, emphasizing their importance in the context of post-transcriptional regulation. However, many lncRNAs related to the development of muscle tissues in domestic animals remain to be characterized.

m^6^A modifications involve the methylation of the amino group at the 6^th^ position of adenine residues in RNA molecules. This reversible epigenetic modification is regulated in a coordinated manner by methyltransferases (writers) and demethylases (erasers) in the nucleus, thereby controlling the overall levels of m^6^A modification and the specific sites that are modified. The m^6^A reader proteins can then recognize these modifications to control the splicing, transport, translation and degradation of the modified RNAs [15–18]. m^6^A modification is catalyzed by methyltransferase complexes, primarily *METTL3*, *METTL14* and *WTAP*. *FTO* and *ALKBH5* are m^6^A demethylases capable of removing the m^6^A modifications from RNA molecules that have been methylated [19]. The m^6^A reader proteins are generally classified into three groups, including (1) YTH domain-containing readers (YTHDC1, YTHDC2, YTHDF1, YTHDF2 and YTHDF3) capable of recognizing transcripts that have undergone m^6^A modification; (2) the 43S translation initiation complex protein eIF3 (eukaryotic initiation factor 3), which modulates translational initiation by binding to the 5′ untranslated region (UTR) of mRNAs that have undergone m^6^A modification [15]; and (3) factors capable of stabilizing mRNAs that have undergone m^6^A modification, including insulin-like growth factor binding proteins 1–3 (IGF2BP1–3) and Prrc2a [20, 21]. A growing body of evidence has attested to the important role that m^6^A modifications play in the coordination of the development of skeletal muscle. In pigs, for instance, whole-transcriptome m^6^A mapping of the prenatal skeletal muscle development process highlighted dynamic shifts in the m^6^A methylome over time, with most impacted genes being closely associated with pathways relevant to skeletal muscle development [22]. In goats, knocking down *FTO* is associated with an increase in *GADD45B* m^6^A levels and a reduction in *GADD45B* mRNA stability during muscle development, ultimately suppressing myogenic differentiation [23]. One recent report highlighted the effects of m^6^A modification during bovine muscle cell development, and *METTL3* and *METTL14* silencing were shown to improve bovine myoblast proliferation while inhibiting the differentiation of these cells [24].

Bohai black cattle, previously referred to as Wudi black cattle, are one of the three most prominent breeds of black cattle globally and are members of the yellow cattle family. In this study, the dynamic profiling of the lncRNA methylome and transcriptome was performed in bovine skeletal muscle samples collected from five stages of development ranging from the newborn stage to 30 months of age. The potential biological importance of lncRNA methylation during development of skeletal muscle was assessed. These findings provide insights into the molecular processes governing ruminants skeletal muscle development, laying a theoretical foundation for future studies on the specific mechanisms that regulate skeletal muscle development and advancing the selection and breeding of Bohai black cattle.

## Materials and methods

### Tissue samples

Samples of skeletal muscle (longissimus dorsi muscle, LDM) were collected from Bohai black cattle at 5 stages of development, including 0, 6, 12, 20 and 30 months postnatally (M0, M6, M12, M20 and M30). At each of these stages, samples were collected from three bulls as biological replicates and stored in liquid nitrogen. 

### MeRIP-seq and RNA-seq library construction

Total RNA was extracted from 15 samples using TRIzol (Cat. 15596-026, Invitrogen, Carlsbad, CA, USA) as directed. A NanoDrop spectrophotometer (NanoDrop ND-1000, NanoDrop Technologies, Wilmington, DE, USA) was used to quantify RNA and check purity. A bioanalyzer (Agilent 2100 Bioanalyzer, Agilent Technologies Inc.*,* Santa Clara, CA, USA) was then used to establish the quality of the isolated RNA, retaining samples with a RIN < 7.0 and confirming these results through denaturing agarose gel electrophoresis. More than 25 μg of total RNA from a particular sample was used to deplete rRNA with an Epicentre Ribo-Zero Gold Kit (Cat. RZH1046, Epicentre, Madison, WI, USA). Purified rRNA-depleted RNA was then fragmented with a Magnesium RNA Fragmentation Module (Cat. E6150, New England Biolabs, Ipswich, MA, USA) at 86 °C for 7 min and incubated with an m^6^A-specific antibody (Cat. 202003, Synaptic Systems, Göttingen, Lower Saxony, GER) in IP buffer (50 mmol/L Tris-HCl, 750 mmol/L NaCl and 0.5% Igepal CA-630) for immunoprecipitation. The precipitated RNA was used to prepare cDNA with SuperScript™ II Reverse Transcriptase (Cat. 1896649, Invitrogen, Carlsbad, CA, USA), which was then used for the preparation of U-labeled second-stranded DNA using *E. coli* DNA polymerase I (Cat. M0209, New England Biolabs, Ipswich, MA, USA), RNase H (Cat. M0297, New England Biolabs, Ipswich, MA, USA) and a dUTP Solution (Cat. R0133, Thermo Fisher Scientific, Waltham, MA, USA). The blunt ends of each strand then had an A-base added to prepare for index adapter ligation, with each adapter harboring a T-base overhand to allow for ligation of the A-tailed DNA fragments. These fragments were ligated to single- or dual-index adapters, after which AMPureXP beads were used for size selection. U-labeled second-stranded DNA was then treated with the heat-labile UDG enzyme (Cat. M0280, New England Biolabs, Ipswich, MA, USA), and ligated products were subjected to PCR amplification (95 °C for 3 min; 8 cycles of 98 °C for 15 s, 60 °C for 15 s, and 72 °C for 30 s; 72 °C for 5 min). The final cDNA library had an average insert size of 300 ± 50 bp. At last, 2 × 150 bp paired-end sequencing (PE150) of these samples was performed using an Illumina Novaseq™ 6000 (LC-Bio Technology Co., Ltd., Hangzhou, China).

### Bioinformatics analyses

Adaptor-containing, low-quality and undetermined reads were removed using Fastp (https://github.com/OpenGene/fastp) with default settings [25]. FastQC (https://www.bioinformatics.babraham.ac.uk/projects/fastqc/) and RseQC (http://rseqc.sourceforge.net/) [26, 27] were used for the sequence validation of IP and input samples, while the read mapping to the *Bos taurus* reference genome (ARS-UCD1.2, Ensemblv107) was performed with HISAT2 (http://daehwankimlab.github.io/hisat2) [28]. The R exomePeak package (http://bioconductor.jp/packages/3.17/bioc/html/exomePeak2.html) was used for peak calling and differential peak analyses, with peak annotation being achieved based on the overlap with gene architecture using the R ANNOVAR package (https://annovar.openbioinformatics.org/en/latest/) [29, 30]. De novo and known motifs were identified with HOMER (http://homer.ucsd.edu/homer/motif), followed by motif localization relative to the peak summit [31]. Transcript and gene expression analyses were conducted using StringTie (https://ccb.jhu.edu/software/stringtie), calculating FPKM values (total exon fragments/mapped reads (millions) × exon length (kb)) to quantify expression levels [32]. lncRNAs considered expressed such that they were retained for subsequent analysis were those with an average FPKM ≥ 0.1 in three samples. The threshold for differential transcript expression was |log_2_ (fold change)| ≥ 0.585 and *P* < 0.05, as determined with the R edgeR package (https://bioconductor.org/packages/release/bioc/html/edgeR.html) [33].

### lncRNA identification

Unqualified sequences were filtered out Cutadapt (https://cutadapt.readthedocs.io/en/stable/) [34], after which HISAT2 (https://daehwankimlab.github.io/hisat2/) [28, 35, 36] was used to remove portions of reads with low quality and to map the remaining reads to the reference genome. StringTie (https://ccb.jhu.edu/software/stringtie) [32, 36, 37] was then used to establish the transcripts, with gffcompare (http://ccb.jhu.edu/software/stringtie/gffcompare.shtml) [38, 39] being used to identify novel transcripts. For this approach, transcripts overlapping with known mRNAs and lncRNAs or transcripts < 200 bp long were screened. Then, CPC0.9-r2 (http://cpc2.cbi.pku.edu.cn) and CNCI2.0 (https://github.com/www-bioinfo-org/CNCI#install-cnci) were used to predict new transcripts with coding potential using the default analytical parameters, retaining those transcripts with a CPC score < 0.5 and a CNCI score < 0 as putative novel lncRNAs. The remaining transcripts with class codes (I, j, o, u, x) were regarded as novel lncRNAs. The known and novel lncRNA datasets were then combined into a final lncRNA dataset for further analysis.

### Weighted gene co-expression network analysis (WGCNA)

An R WGCNA package was used to perform a WGCNA using the provided tutorials. Euclidean distances calculated using gene expression data and integrated with growth and development-related parameters were used to cluster the 15 cattle LDM samples in this study. Network topology analyses ensured a scale-free topology, using a soft-thresholding power of 5. A dynamic tree-cutting algorithm with parameters minModuleSize at 30 and mergeCutHeight at 0.25 identified four modules. The eigengene (defined as the first component expression of genes in that module) was determined, and correlations between these eigengenes and cattle body weight, withers height, hip height, body length, chest circumference, abdominal circumference and cannon bone circumference were assessed. Genes exhibiting a high degree of connectivity within the established modules were regarded as hub genes.

### Functional enrichment analyses

The potential functional roles of the analyzed lncRNAs were explored by selecting expressed mRNAs within 100 kb as putative *cis-*target genes [40]. Significantly differentially expressed lncRNAs and mRNAs were identified based on a threshold of |log_2_FC|> 1 and *P* < 0.05. mRNAs within 100 kb upstream or downstream of lncRNAs were identified as potential target genes. Finally, these mRNAs were used to perform Gene Ontology (GO) and Kyoto Encyclopedia of Genes and Genomes (KEGG) pathway enrichment analyses using OmicStudio (https://www.omicstudio.cn/tool), with terms having *P* < 0.05 considered statistically significant.

### MeRIP-qPCR

A NEB/EpiMark® N^6^-Methyladenosine Enrichment Kit (Cat. E1610S, New England Biolabs, Ipswich, MA, USA) was used for MeRIP analyses establishing individual transcript m^6^A modification status. Briefly, 150 μg of total RNA from pretreated samples was randomly fragmented to yield < 100 nucleotide fragments, which were then immunoprecipitated using magnetic beads that had been coated with 10 μg of anti-m^6^A (Cat. 202003, Synaptic Systems, Göttingen, GER). The precipitated m^6^A-modified fragments of RNA were then eluted with N^6^-methyladenosine 5′-monophosphate sodium salt (6.7 mmol/L), after which MeRIP-qPCR analyses were performed with appropriate primers developed with MeRIP-Seq data and the motif-dependent m^6^A site predictor SRAMP (http://www.cuilab.cn/sramp) (Table S3). Relative m^6^A enrichment was normalized against the input RNA as follows: %input = 1/10 × 2Ct [IP] – Ct [input].

### qPCR

TRIzol (Cat. 15596-026, Invitrogen, Carlsbad, CA, USA) was used to extract total RNA from PSCs and tissue samples, and a PrimeScript™ RT reagent Kit with gDNA Eraser (Cat. RR047A; Takara, Shiga, Japan) was used to prepare cDNA that was subsequently used for qPCR analyses on an ABI 2720 Thermal Cycler (Thermo Fisher Scientific, Waltham, MA, USA) with PowerUp SYBR Green Master Mix (Cat. A25742, Thermo Fisher Scientific, Waltham, MA, USA). Relative expression was established with the Ct (2^−ΔΔCT^) method. Primers for selected genes (Table S4) were designed using the NCBI Primer BLAST software. Primer pairs were selected to minimize non-specific amplification, with high ΔG values to avoid self or pair dimers and hairpin formations. Primer pairs were designed to amplify products spanning exon-exon junctions to avoid annotated variants from public SNP databases (https://www.ncbi.nlm.nih.gov/snp/). After the primer design, the predicted product was BLAST-searched against the bovine database to ensure the specificity of the primers.

### Statistical analyses

qPCR data were analyzed using SAS (version 9.2; SAS Institute, Cary, NC, USA). GraphPad Prism (version 6.0; GraphPad Software, San Diego, CA, USA) was used to prepare figures. Data are presented as mean ± standard error of the mean (SEM), with significance set at *P* < 0.05.

## Results

### Characterization of the dynamic RNA methylome and transcriptome during skeletal muscle development

**Fig. 1:**
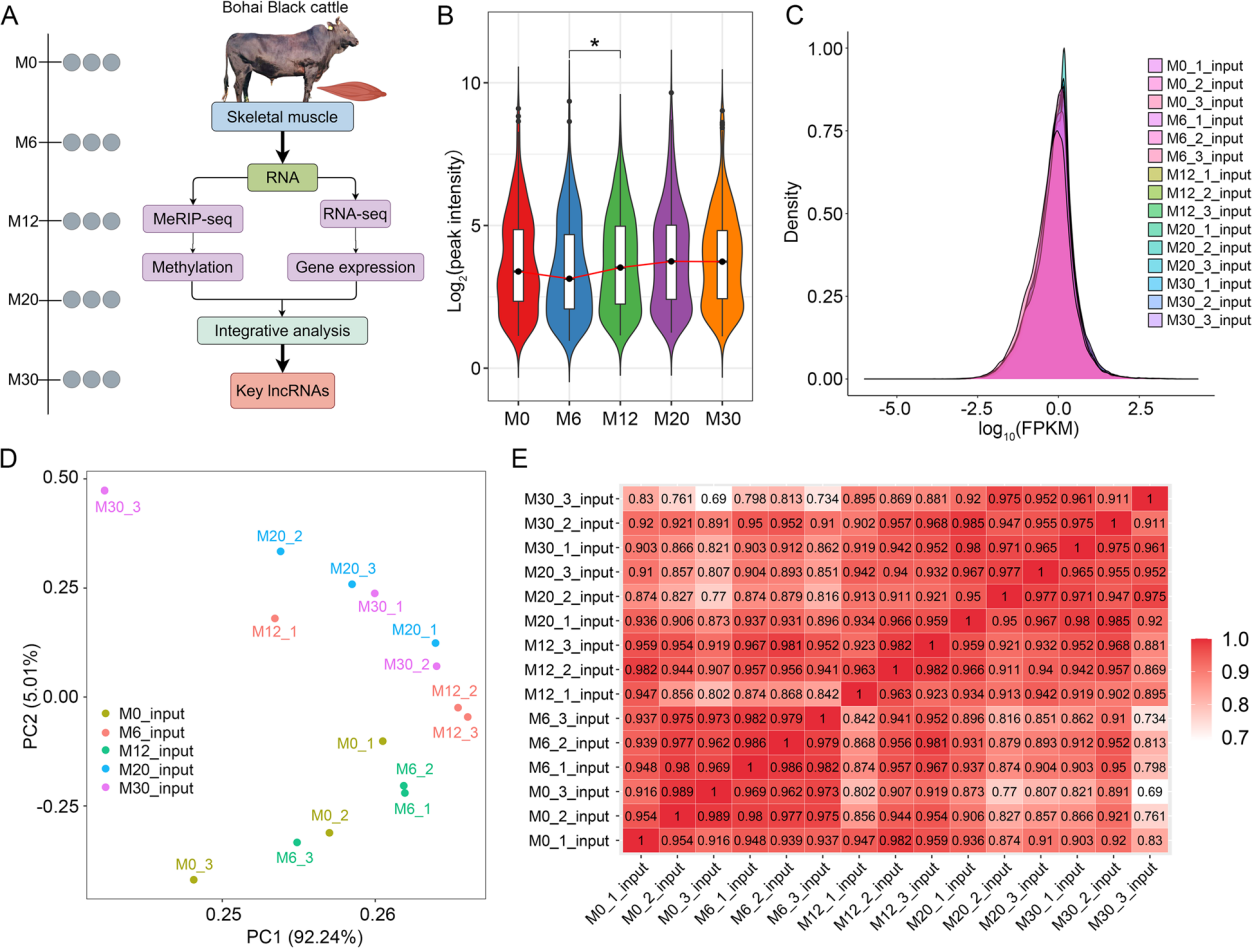
RNA methylome and transcriptomic profiles during skeletal muscle development. **A** The experimental workflow for profiling the RNA methylome and transcriptome across five stages of skeletal muscle development in cattle (illustration by Figdraw, ID: ISIIO07141). **B** Methylation levels of lncRNAs at each developmental stage. **C** Distribution map of lncRNA expression. **D** Principal component analysis (PCA) of the samples. **E** Pearson correlation analysis of RNA-seq data for each sample pair, accompanied by hierarchical clustering. Darker colors represent stronger correlations between samples. ^*^P < 0.05

In an effort to systematically examine the regulatory and functional importance of RNA methylation in the context of skeletal muscle development, the dynamic changes in the RNA methylome and transcriptome were characterized via MeRIP-seq and RNA-seq in skeletal muscle tissue samples over five stages of development (Fig. 1A). MeRIP-seq analyses yielded 1,121,349,968 raw reads covering 160.43 Gb of sequence (Table S1), while RNA-seq analyses yielded 1,266,898,132 raw reads covering 181.69 Gb of sequence (Table S2). Clean MeRIP-seq and RNA-seq reads had average mapping rates of 95.64% (94.03%–96.55%) and 96.46% (95.84%–96.88%) to the ARS-UCD1.2 reference genome (Ensemblv107), respectively.

Dynamic changes in lncRNA m^6^A methylation were assessed over the skeletal muscle developmental process using methylomic sequencing data, revealing the lowest and highest levels of lncRNA methylation in the M6 and M20 samples, respectively (Fig. 1B). Transcriptomic data showed high intersample correlation, with no clear differentiation among groups based on Pearson's correlation coefficients, principal component analyses and density curves (Fig. 1C–E).

### Developing skeletal muscle exhibits dynamic changes in lncRNA methylation

**Fig. 2:**
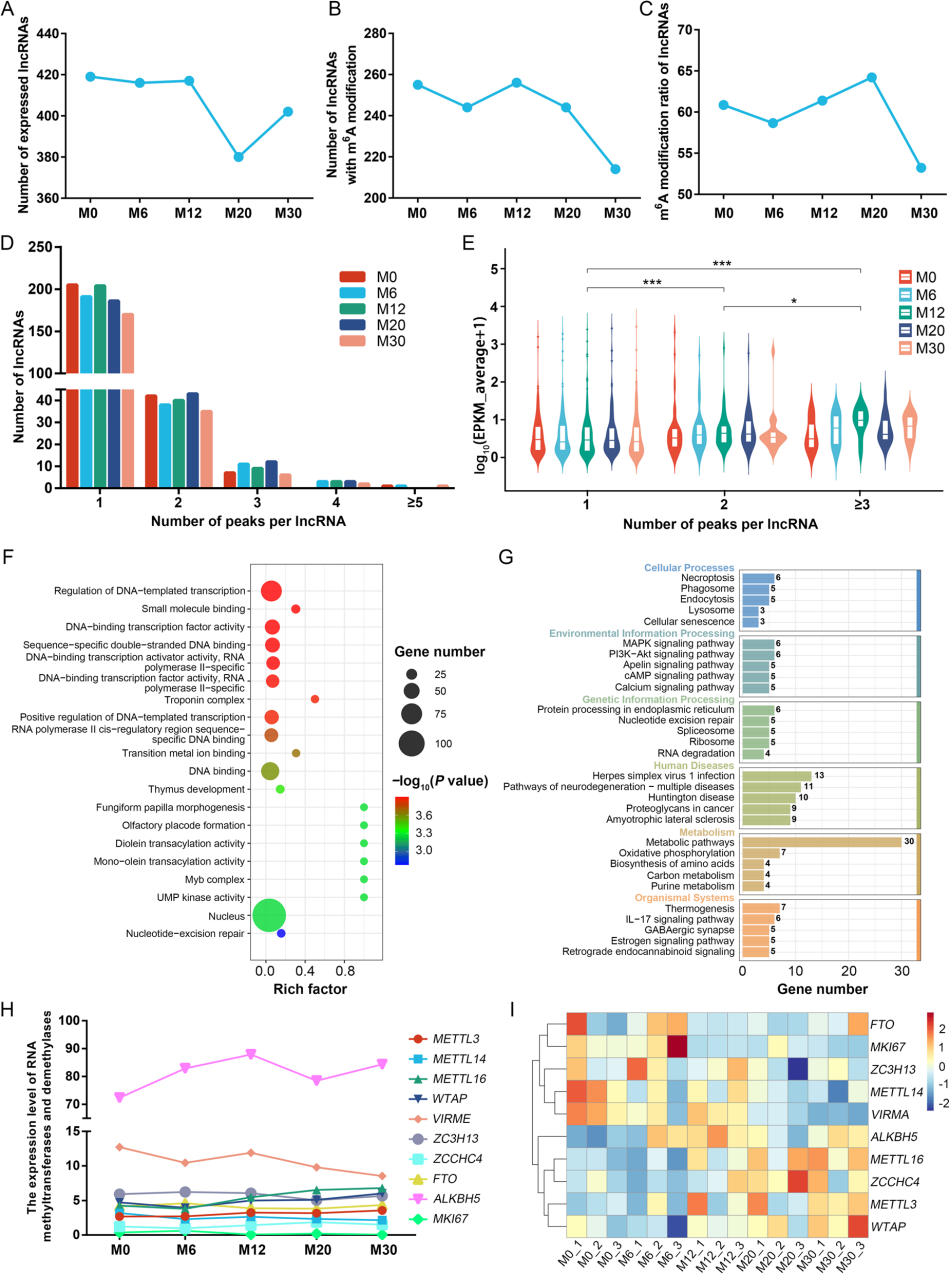
Global lncRNA methylation dynamics during skeletal muscle development. **A** Number of lncRNAs expressed at each developmental stage. **B** Number of m^6^A-modified lncRNAs across stages. **C** Proportion of lncRNAs with m^6^A modifications at each stage. **D** Distribution of peak counts in m^6^A-modified lncRNAs. **E** Correlations between lncRNA expression levels and m^6^A peak counts. **F–G** Gene Ontology (GO) (**F**) and KEGG pathway (**G**) enrichment analyses of lncRNAs with single m^6^A peaks. **H–I** RNA-seq-based expression analysis of RNA methyltransferase and demethylase genes (**H**) and their corresponding heatmaps (**I**). ^*^P < 0.05, ^***^P < 0.01

Dynamic changes in the lncRNA methylome were examined throughout skeletal muscle development. We detected 419, 416, 417, 380 and 402 lncRNAs expressed in the M0, M6, M12, M20 and M30 groups respectively (Fig. 2A), of which 255, 244, 256, 244 and 214 were respectively m^6^A-modified (Fig. 2B), for respective m^6^A modification proportions of 60.86%, 58.65%, 61.39%, 64.21% and 53.23% (average: 59.67%, Fig. 2C). These dynamic changes in lncRNA m^6^A modification over the developmental process also coincided with a general reduction in the overall level of RNA methylation, declining from 60.86% at the newborn (M0) stage to 53.23% at 30 months of age (M30).

Numbers of m^6^A peaks for each lncRNA were analyzed, revealing that most had 1–2 such peaks (Fig. 2D). Correlation analyses showed that lncRNAs with three or more m^6^A peaks had higher expression levels than those with one or two peaks (Fig. 2E). The relationship between lncRNA function and m^6^A peak numbers was also assessed. As lncRNAs cannot code for proteins under normal circumstances, they function primarily through their effects on gene targets. *Cis*-target genes of these lncRNAs were thus leveraged in an effort to understand their potential functions through GO and KEGG enrichment analyses of these genes. These analyses showed no significant differences in enriched pathways or functions based on m^6^A peak numbers. These *cis*-target genes were enriched in pathways such as transcriptional regulation, DNA binding, RNA polymerase II transcriptional function, cAMP, calcium signaling, Apelin, cGMP-PKG and TNF signaling (Fig. 2F and G, Fig. S1).

Changes in RNA methylation status are under the dynamic control of RNA methyltransferases and demethylases [41–44]. RNA-seq data were thus used to assess the expression of these genes. The most highly expressed methyltransferase and demethylase genes identified in this study were *VIRMA* and *ALKBH5*, respectively (Fig. 2H). *VIRMA* levels were gradually downregulated with the progression of skeletal muscle development, whereas *ALKBH5* levels were upregulated. These trends coincided with decreased muscle cell proliferation as indicated by the reduced expression of the proliferation biomarker *MKI67* throughout development (Fig. 2I). These results indicated that the machinery responsible for maintaining methylation and generating de novo methylation were both downregulated with the development of skeletal muscle.

### Dynamic changes in differentially methylated peaks and lncRNAs are related to skeletal muscle development

**Fig. 3:**
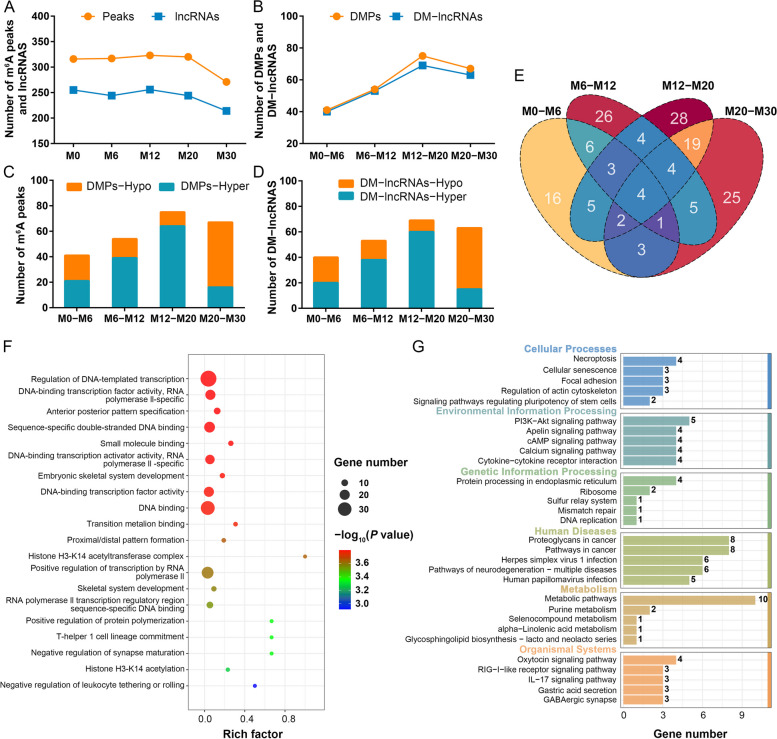
Differential methylation of peaks and lncRNAs in skeletal muscle. **A** Number of m^6^A peaks and methylated lncRNAs at specific developmental stages. **B** Count of DMPs and DM-lncRNAs. **C–D** Count of hypomethylated and hypermethylated DMPs (**C**) and DM-lncRNAs (**D**). **E** Venn diagram showing overlaps among DM-lncRNAs. **F–G** GO (**F**) and KEGG (**G**) enrichment analyses of *cis*-target genes associated with DM-lncRNAs

Initial analysis identified 316, 317, 323, 320 and 271 m^6^A peaks across the five stages of development (M0, M6, M12, M20 and M30) that were respectively associated with 255, 244, 256, 244 and 214 methylated lncRNAs (Fig. 3A). To fully explore the changes in m^6^A methylation status over the process of skeletal muscle development, pairwise comparisons were employed to analyze m^6^A peaks and methylated lncRNAs between consecutive stages. For the M0–M6, M6–M12, M12–M20 and M20–M30 comparisons, 41, 54, 75 and 67 differentially methylated peaks (DMPs), respectively, were identified that were associated with 40, 53, 69 and 63 differentially methylated lncRNAs (DM-lncRNAs) (Fig. 3B). A total of 225 DM-lncRNAs were identified, with 151 retained for further analysis after duplicate removal. The four control groups had 21, 39, 64 and 16 upregulated DMPs (20, 38, 60 and 15 DM-lncRNAs) and 20, 15, 11 and 51 downregulated DMPs (20, 15, 9 and 48 DM-lncRNAs) (Fig. 3C and D). Only a small number of common lncRNAs were detected among these DM-lncRNAs, suggesting that the development of skeletal muscle is characterized by very dynamic shifts in m^6^A levels (Fig. 3E). Potential DM-lncRNA functions in this context were next evaluated through GO and KEGG enrichment analyses of the 224 *cis*-target genes associated with these DM-lncRNAs. In GO analyses, these *cis*-target genes were primarily enriched in the regulation of DNA-templated transcription and DNA binding biological processes (Fig. 3F). KEGG pathway analyses indicated that these *cis*-target genes were primarily enriched in metabolic and human disease-related pathways, in addition to the PI3K-Akt, cAMP and calcium signaling pathways relevant to skeletal muscle development (Fig. 3G). Together, these analyses revealed many DM-lncRNAs expressed in the skeletal muscle in different stages of development, emphasizing the roles that these lncRNAs may play in this developmental process.

### Skeletal muscle development is characterized by dynamic changes in the lncRNA transcriptome

**Fig. 4:**
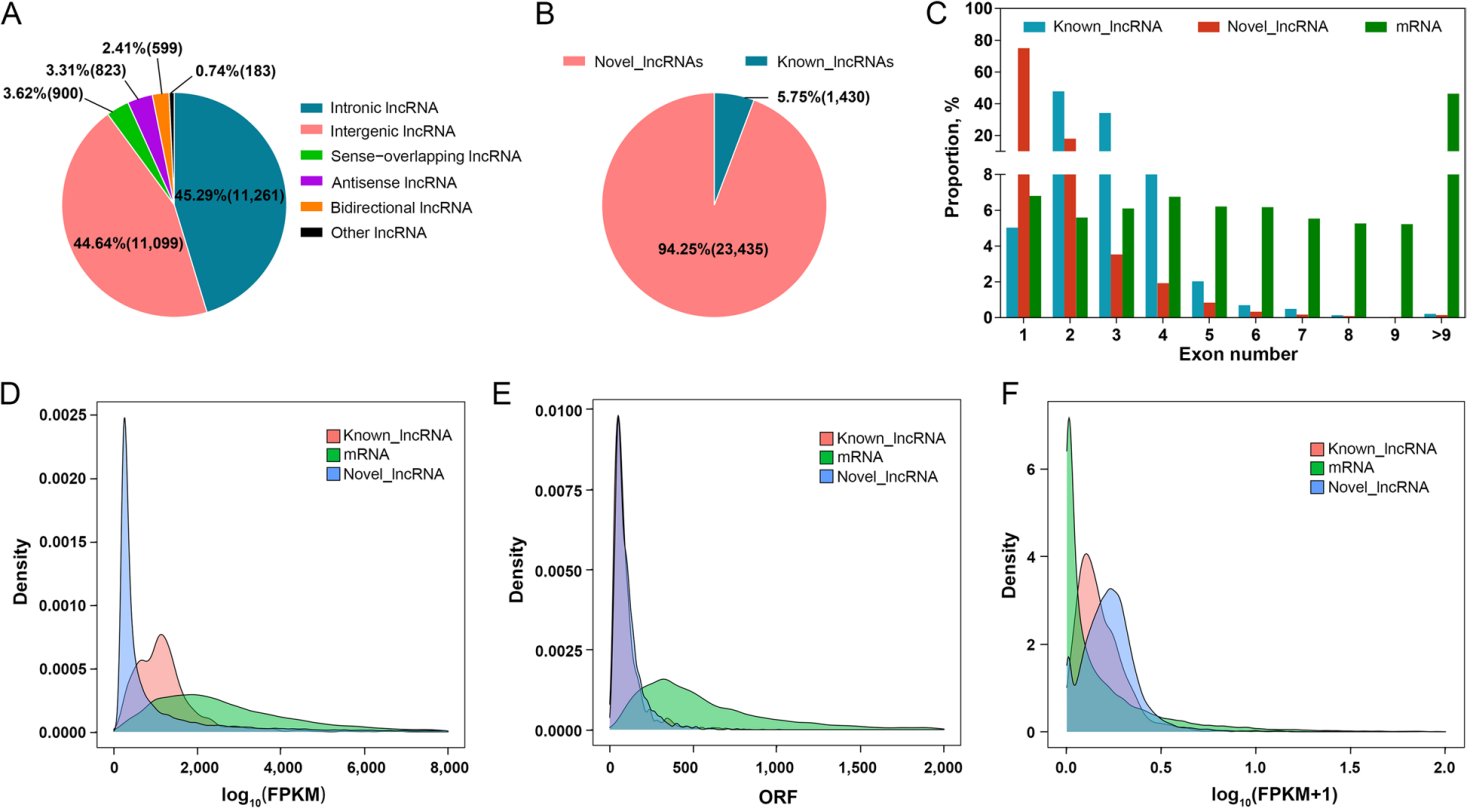
Characterization of lncRNAs identified during skeletal muscle development. **A** Proportion of various lncRNA types identified in this study. **B** Classification of identified lncRNAs. **C–E** Distribution of exon numbers (**C**), transcript lengths (**D**) and open reading frame (ORF) lengths (**E**) in lncRNAs and mRNAs. **F** Expression level distribution (log_10_(FPKM + 1)) of lncRNAs and mRNAs

Transcriptomic analysis identified 24,865 lncRNAs, classified as intronic 45.29% (11,261), intergenic 44.64% (11,099), sense-overlapping 3.62% (900), antisense 3.31% (823) and bidirectional 2.41% (599) (Fig. 4A). These included 23,435 novel lncRNAs (Fig. 4B). To better characterize these novel lncRNAs, comparisons with gene structure and expression were performed among novel lncRNAs, annotated lncRNAs and mRNAs. Novel and annotated lncRNAs showed similar features, including shorter transcript lengths, fewer exons and shorter ORFs than mRNAs. Most of these novel and annotated lncRNAs had expression levels from 0–0.5, whereas most mRNAs had expression values in the 0–0.25 range (Fig. 4C–F).

**Fig. 5:**
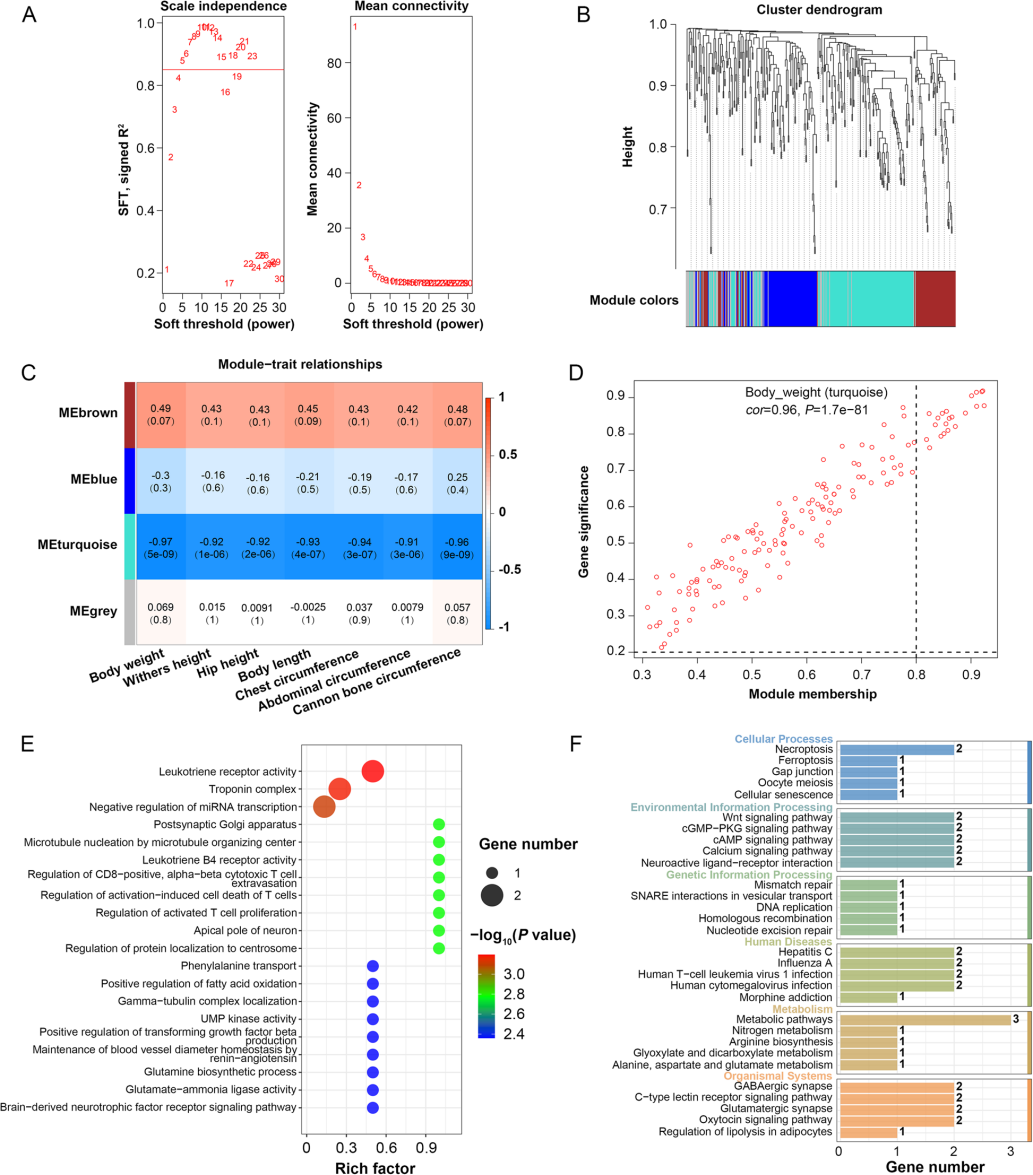
Weighted Gene Co-expression Network Analysis (WGCNA) of annotated lncRNAs in skeletal muscle development. **A** Scale-free network coefficient (*R*^*2*^) analysis for soft thresholding (*β*) and corresponding mean connectivity. The red line indicates *R*^*2*^ = 0.85, with *β* = 5. **B** Cluster dendrogram based on topological overlap dissimilarity, identifying four modules. **C** Heatmap showing correlations between module eigengenes and traits associated with growth and development. Each cell includes the correlation coefficient and *P* value. **D** Scatter plot of module eigengenes, highlighting the turquoise module, where hub lncRNAs were associated with body weight (module membership = 0.8, gene significance = 0.2). **E–F** GO (**E**) and KEGG (**F**) enrichment analyses of hub lncRNAs

Transcriptomic data were used in a WGCNA to identify correlations between annotated lncRNAs and traits relevant to cattle development. A network with scale-free topology was achieved at *β* = 5, with a scale independence value of 0.85 and lower levels of mean connectivity (Fig. 5A). lncRNAs showing similar expression dynamics were grouped into modules via hierarchical clustering, with a height threshold of 0.25, merging highly similar modules until ultimately obtaining a final set of four modules (Fig. 5B). Correlations between these modules and cattle traits were then assessed, revealing that the turquoise model was the most strongly negatively correlated with all of these traits (*r* = −0.97 to −0.91, *P* = 9e-09 to 1e-06) (Fig. 5C). This suggests that genes in the turquoise module may regulate these critical growth-related traits. Hub lncRNAs within the turquoise module were then used for functional enrichment analysis, and correlations between module membership and all growth and development-related traits were established. A preliminary assessment led to the selection of 21 hub lncRNAs from this module (Fig. 5D, Fig. S2). GO enrichment analyses of *cis*-target genes associated with these 21 lncRNAs were then performed, revealing their enrichment in terms including leukotriene receptor activity, troponin complex and negative regulation of miRNA transcription (Fig. 5E). They were also enriched in the KEGG Wnt, cGMP-PKG, cAMP and calcium signaling pathways (Fig. 5F). These 21 hub lncRNAs may thus serve as important mediators of the dynamic regulation necessary for appropriate skeletal muscle development.

### Establishment of key skeletal muscle development-related lncRNAs

**Fig. 6:**
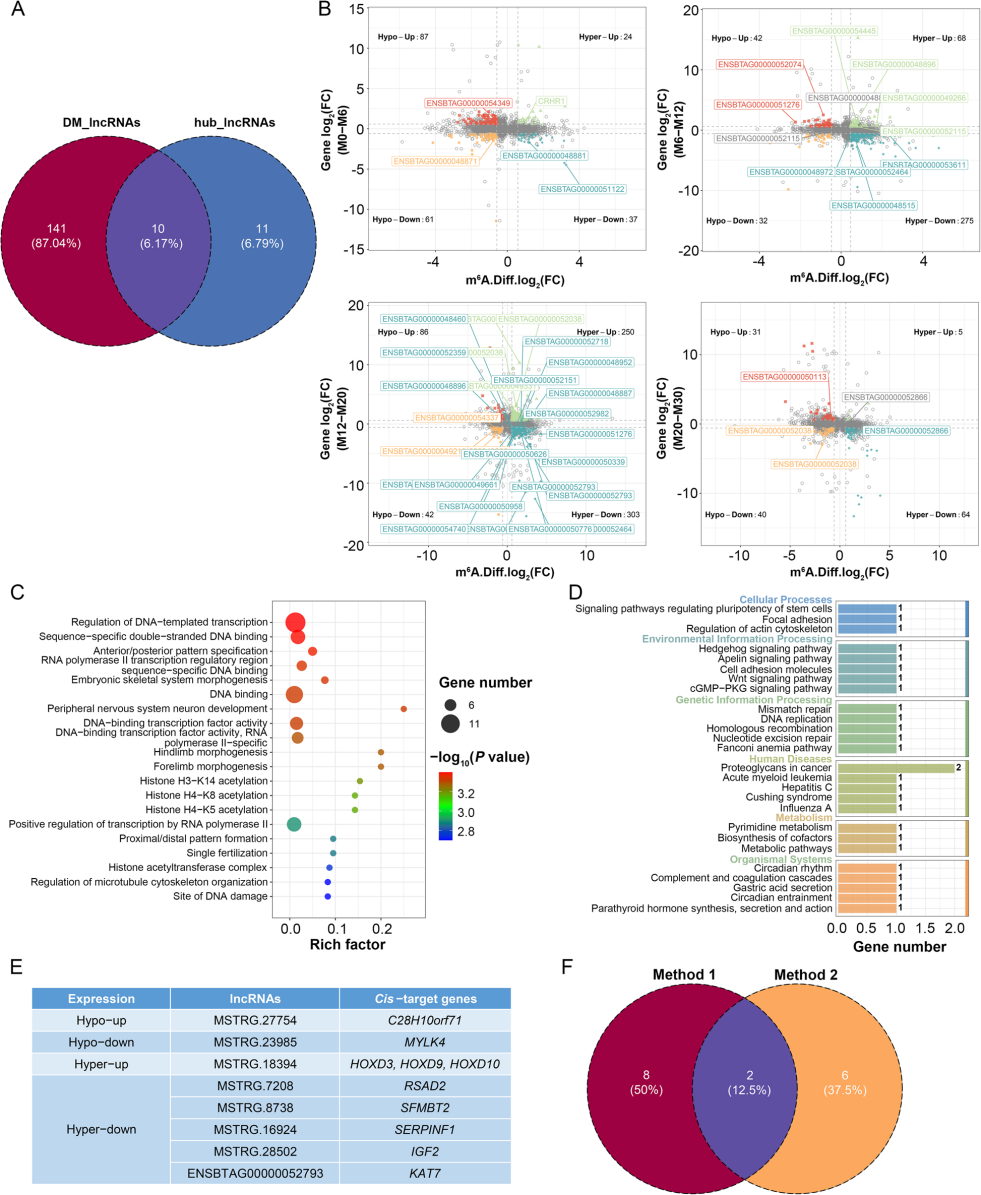
Selection of key lncRNAs associated with skeletal muscle development. **A** Venn diagram illustrating the overlap between DM-lncRNAs and hub lncRNAs. **B** A four-quadrant diagram showing 45 dme-lncRNAs identified in M0**–**M6, M6–M12, M12–M20 and M20–M30 comparisons. **C–D** GO (**C**) and KEGG (**D**) enrichment analyses for *cis*-target genes of dme-lncRNAs. **E** Overview of lncRNAs and their associated *cis*-target genes. **F** Venn diagram showing overlap between Methods 1 and 2

Two methods were next leveraged for the identification of key lncRNAs during the development of skeletal muscle. In the initial approach (Method 1), the intersection of the 151 DM-lncRNAs and 21 hub lncRNAs identified above yielded a list of 10 shared genes established as candidate lncRNAs (Fig. 6A).

In the second approach (Method 2), a conjoint analysis of the MeRIP-seq and RNA-seq datasets was performed. Pairwise comparisons identified 5, 10, 27 and 3 lncRNAs that were both differentially m^6^A modified and differentially expressed (dme-lncRNAs). Of the 45 total dme-lncRNAs, 12 upregulated ones were significantly methylated, with 9 hyper-methylated (hyper-up) and 3 hypo-methylated (hypo-up). Meanwhile, 33 down-regulated lncRNAs were significantly methylated, with 27 hyper-methylated (hyper-down) and 6 hypo-methylated (hypo-down) (Fig. 6B). Following duplicate lncRNAs removal, 36 dme-lncRNAs were retained as candidate lncRNAs associated with 78 putative *cis*-target genes. GO enrichment analyses of these genes indicated that they were enriched in the DNA-templated transcription and DNA binding terms (Fig. 6C). Furthermore, three *cis*-target genes associated with two dme-lncRNAs (MSTRG.27754 and MSTRG.18394) were enriched in pathways related to skeletal muscle development. *Cis*-target genes for MSTRG.18394 were identified as the *HOX* family members *HOXD3*, *HOXD9* and *HOXD10*, all of which control key developmental processes [45–47]. KEGG pathway analyses of the *cis*-target genes of these lncRNAs also indicated that they are enriched in muscle development-related pathways including the Hedgehog, Wnt and cGMP-PKG signaling pathways (Fig. 6D). In total, 10 *cis*-target genes associated with 8 dme-lncRNAs (MSTRG.27754, MSTRG.8738, MSTRG.23985, MSTRG.18394, MSTRG.7208, MSTRG.16924, MSTRG.28502 and ENSBTAG00000052793) were analyzed. This approach yielded 8 dme-lncRNAs that may help shape skeletal muscle development (Fig. 6E). In conclusion, 16 total candidate lncRNAs were identified by combining results derived from these two analytical approaches (Fig. 6F).

### MeRIP-qPCR and qPCR validation of study results

**Fig. 7:**
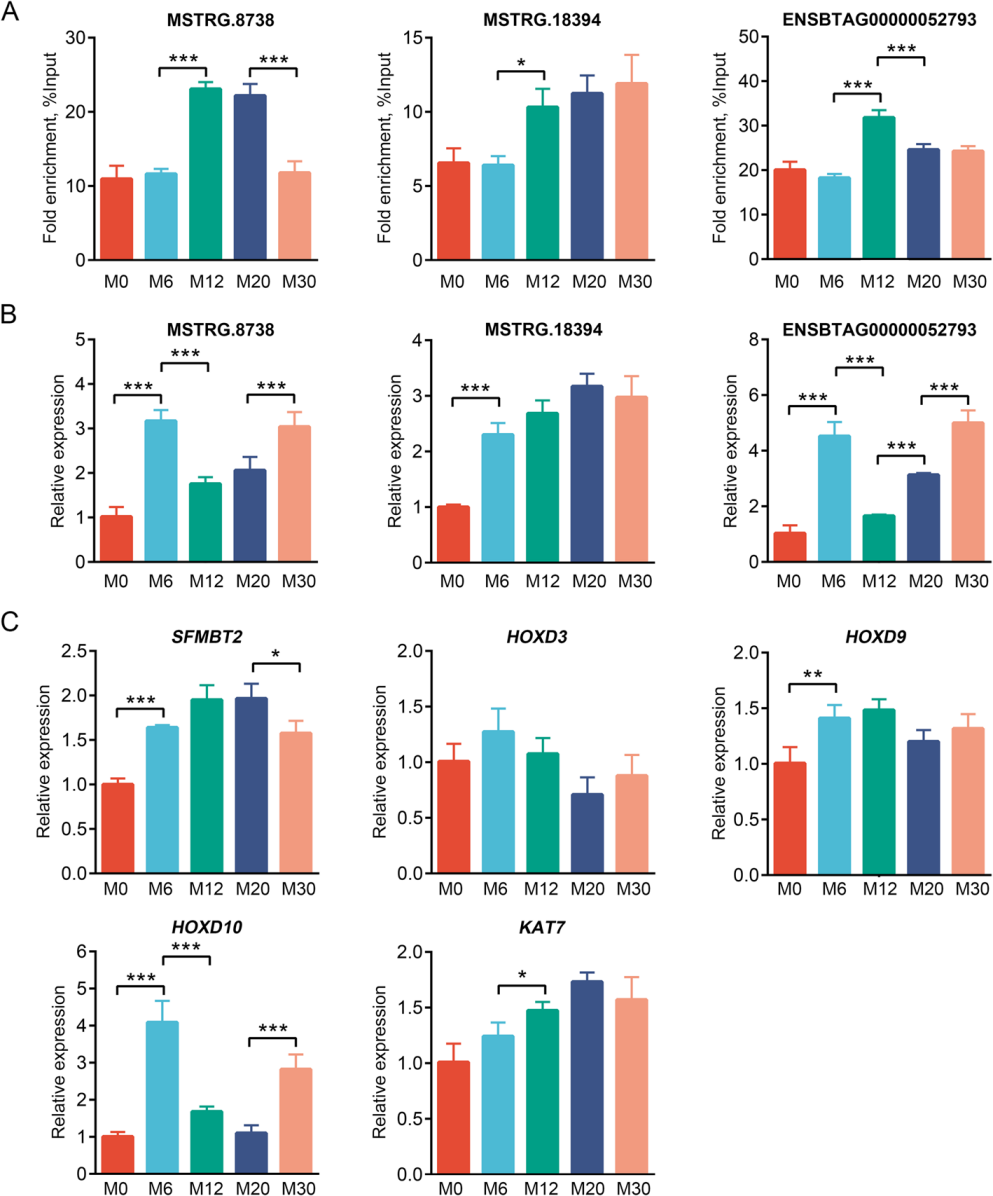
Validation of findings using MeRIP-qPCR and qPCR. **A–B** Validation of three dme-lncRNAs using MeRIP-qPCR (**A**) and qPCR (**B**). **C** Validation of five *cis*-target genes using qPCR. Relative expression levels were normalized to *GAPDH*. Data are presented as mean ± SD for three independent biological replicates. ^*^P < 0.05, ^**^P < 0.01, ^***^P < 0.001

Three dme-lncRNAs (MSTRG.8738, MSTRG.18394 and ENSBTAG00000052793) were selected for MeRIP-qPCR and qPCR to validate their methylation and expression levels (Fig. 7A and B). The results showed that these three dme-lncRNAs had m^6^A enrichment across five developmental stages. The m^6^A methylation levels of MSTRG.8738 and ENSBTAG00000052793 were significantly upregulated, and expression levels were significantly downregulated in M12 samples compared to M6 samples. The m^6^A methylation level of MSTRG.18394 was significantly upregulated, and the expression level showed an upward trend in M12 samples compared to M6 samples.

The expression levels of the *cis*-target genes linked to these three dme-lncRNAs were also analyzed. Compared with M6, the *SFMBT2* expression level in M12 samples showed an upward trend. However, *HOXD10* expression was significantly downregulated, while *KAT7* expression was significantly upregulated (Fig. 7C). These results confirm the m^6^A modification status of specific lncRNAs in Bohai black bovine skeletal muscle.

## Discussion

This study presents dynamic genome-wide transcriptomic and methylomic maps for five stages of skeletal muscle development. This is the first reported effort to systemically profile the lncRNA m^6^A methylome in bovine skeletal muscle to the best of our knowledge. While it is important to note that the multi-omics analyses in this study relied on the use of bulk tissue instead of single muscle fibers such that the results may have been influenced by differences in fiber time composition and the populations of non-muscle cells present over the course of development, such forms of bias have been suggested to have minimal confounding effects on study conclusions [48]. These datasets are thus comprehensive tools for efforts to understand the roles that lncRNA methylation plays in the development of skeletal muscle.

In addition to being ubiquitously present among mRNA transcripts, m^6^A methylation is closely tied to the control of gene expression [49]. lncRNA m^6^A methylation has been a significant focus of research. Here, MeRIP-seq identified several m^6^A peaks in lncRNAs from bovine skeletal muscle, with results validated by MeRIP-qPCR analyses. The m^6^A methylation of mRNAs can impact their transport, splicing, translation and stability [15]. Similarly, the m^6^A methylation of lncRNAs can affect expression levels thereof. *THAP7-AS1*, for instance, can be transcriptionally induced by SP1 and m^6^A modified by *METTL3*, whereupon it can support oncogenesis by promoting NLS interactions with importin α1 and enhancing nuclear CUL4B protein entry, repressing miR-22-3p and miR-320a transcription [50]. The stability of the lncRNA *DIAPH1-AS1* can also be improved by m^6^A modification mediated by *WTAP* through a pathway dependent on *IGF2BP2*, whereupon it functions as a molecular adaptor capable of promoting the formation of the MTDH-LASP1 complex and upregulating LASP1 to facilitate the growth and metastasis of nasopharyngeal carcinoma [51].

Approximately 59.67% of lncRNAs showed m^6^A modifications, with levels declining during development, likely due to reduced *VIRMA* expression and increased *ALKBH5* expression. The m^6^A modification of Traf6 was predicted to regulate translation, with no effect observed for transcripts with 1–2 m^6^A peaks, while transcripts with 3 peaks activated the YTHDF1 regulatory mechanism [52]. In this analysis, lncRNAs with three or more m^6^A peaks showed significantly higher expression levels than those with one or two peaks. The mechanistic basis for this observation is uncertain and warrants further research.

RNA-seq analyses of LDM samples from Bohai black cattle at five developmental stages (0, 6, 12, 20 and 30 months) characterized transcriptomic dynamics with high confidence [53]. Novel lncRNAs identified herein were consistent with previous reports regarding length, exon numbers and ORF length [54–58]. Most of the novel and annotated lncRNAs had expression values in the 0–0.5 range. However, most mRNAs showed expression values in the 0–0.25 range, differing from previous findings [54–58].

Two approaches were subsequently employed to identify specific lncRNAs that shape the development of skeletal muscle. Initially, transcriptomic data were analyzed using WGCNA to identify correlations between annotated lncRNAs and developmental traits, resulting in a list of 21 putative hub lncRNAs. The intersection of these 21 hub lncRNAs with 151 DM-lncRNAs identified 10 overlapping candidate lncRNAs. The second approach integrated MeRIP-seq and RNA-seq datasets to identify 36 dme-lncRNAs. *Cis*-target gene analyses have provided valuable insights into lncRNA functions [56]. Accordingly, 78 *cis*-target genes associated with these dme-lncRNAs were herein identified and used to conduct additional functional enrichment analyses. This strategy ultimately identified 8 putative dme-lncRNAs that may influence skeletal muscle development. These two methods identified 16 lncRNAs as final candidates, representing potential regulators of skeletal muscle formation.

MeRIP-qPCR and qPCR were finally used to determine the methylation and gene expression levels of three dme-lncRNAs (MSTRG.8738, MSTRG.18394 and ENSBTAG00000052793) and their *cis*-target genes. In comparison to M6, the methylation level of MSTRG.8738 in M12 was significantly upregulated, and the expression level was significantly downregulated, while the expression of its *cis*-target gene *SFMBT2* demonstrated an increasing trend. These results suggest that increased m^6^A methylation inhibited MSTRG.8738 expression while elevating *SFMBT2* expression, contributing to skeletal muscle development. Nevertheless, the exact mechanism by which MSTRG.8738, with m^6^A modification and its *cis*-target gene *SFMBT2,* regulates myoblast proliferation and differentiation remains unclear and warrants further investigation. Compared with M6, MSTRG.18394 methylation level in M12 was significantly upregulated, and the expression level showed an upward trend, whereas the expression level of its *cis*-target gene *HOXD10* was significantly downregulated. These results indicate that increased m^6^A methylation enhanced MSTRG.18394 expression while inhibiting *HOXD10* expression, supporting skeletal muscle growth and development. The 39 *HOX* genes in mammals are organized into four clusters labeled A through D. These genes can be categorized into 13 paralogous groups (1–13) according to their sequence similarities and positions within the clusters [59, 60]. For the proper growth and skeletal design of tetrapod limbs, *HOX* genes are needed, especially the *HOXA* and *HOXD* clusters, which are vital for the development of both forelimbs and hindlimbs [61, 62]. In the limb development of mice, Shh expression is primarily driven by the genes *HOXA9, 10, 11* and *HOXD9, 10, 11* [63]. Studies indicate that Shh is vital for the early induction of the myogenic determination genes *Myf5* and *MyoD* in epaxial somite cells, which lead to the formation of deep back muscle progenitors [64]. Compared with M6, ENSBTAG00000052793 methylation level in M12 was significantly upregulated, and the expression level was downregulated, while the expression level of its *cis*-target gene *KAT7* was significantly upregulated. These findings suggest that increased m^6^A methylation inhibited ENSBTAG00000052793 expression and increased *KAT7* expression, facilitating skeletal muscle growth and development. Previous studies have shown that lncRNA ADAMTS9-AS is competitively bound to miR-185-5p to upregulate *KAT7* and thus inhibit cardiomyocyte hypertrophy [65]. Another investigation found that *circFoxo3* alleviated myocardial ischemia/reperfusion injury by reducing autophagy, achieved by inhibiting *HMGB1* through the suppression of *KAT7* in myocardial infarction [66]. The three genes identified in this study are potential candidates for regulating skeletal muscle development. Further investigation is required to shed light on how these genes regulate and influence skeletal muscle development.

## Conclusions

In conclusion, this study analyzed the expression and m^6^A methylation profiles of lncRNAs linked to skeletal muscle development, ultimately revealing 16 m^6^A-modified lncRNAs that may play a key regulatory role during this process. These findings may offer evidence for future studies to clarify the mechanistic functions of these m^6^A-modified lncRNAs, providing an opportunity for more comprehensive analyses of the epigenetic modification of RNA during skeletal muscle development.

## Supplementary Information

Additional file 1: Table S1. Sequencing statistics summary of the methylome data.

Additional file 2: Table S2. Sequencing statistics summary of the transcriptome data.

Additional file 3: Table S3. Primers used for MeRIP-qPCR experiments.

Additional file 4: Table S4. Primers used for qPCR experiments.

Additional file 5: Fig. S1. GO and KEGG enrichment analyses of lncRNAs with two or more m^6^A peaks. A GO enrichment analyses of lncRNAs with two m^6^A peaks. B KEGG enrichment analyses of lncRNAs with two m^6^A peaks. C GO enrichment analyses of lncRNAs with 3 + m^6^A peaks. D KEGG enrichment analyses of lncRNAs with 3 + m^6^A peaks.

Additional file 6: Fig. S2. Scatter plots of module eigengenes. A–F The genes of the turquoise module in the upper right were chosen as hub lncRNAs associated with withers height (A), hip height (B), body length (C), chest circumference (D) abdominal circumference (E) and cannon bone circumference (F) (module membership = 0.8, gene significance = 0.2).

## Data Availability

The datasets generated and analyzed during the current study are not publicly available. They can be made available from the corresponding author upon reasonable request.

## Abbreviations

ALKBH5: AlkB homolog 5
ceRNA: Competing endogenous RNA
dme-lncRNAs: Differentially m^6^A modified and differentially expressed lncRNAs
DM-lncRNAs: Differentially methylated lncRNAs
DMP: Differentially methylated peaks
FTO: Fat mass and obesity related
GO: Gene Ontology
KEGG: Kyoto Encyclopedia of Genes and Genomes
IGF2BP1–3: Insulin-like growth factor binding proteins 1–3
LDM: Longissimus dorsi muscle
lncRNA: Long non-coding RNA
MeRIP-qPCR: Methylated RNA immunoprecipitation-real-time quantitative PCR
MeRIP-seq: Methylated RNA immunoprecipitation sequencing
MKI67: Marker of proliferation Ki-67
METTL3: Methyltransferase-like 3
METTL14: Methyltransferase-like 14
m^6^A: N^6^-methyladenosine
Prrc2a: Proline-rich coiled-coil2A
qPCR: Real-time quantitative PCR
RNA-seq: RNA sequencing
UTR: Untranslated region
VIRMA: Vir like N^6^-methyladenosine methyltransferase associated protein
WGCNA: Weighted gene co-expression network analysis
WTAP: Wilms tumor 1-associating protein
YTHDC1–2: YTH domain-containing 1–2
YTHDF1–3: YTH-domain family 1–3
