# Supplementary figures and images for: Comprehensive characterization of lncRNA N6-methyladenosine modification dynamics throughout bovine skeletal muscle development
Source: J Anim Sci Biotechnol. 2025 Mar 6;16:36. doi: 10.1186/s40104-025-01164-2 (PMC11884139; doi:10.1186/s40104-025-01164-2)

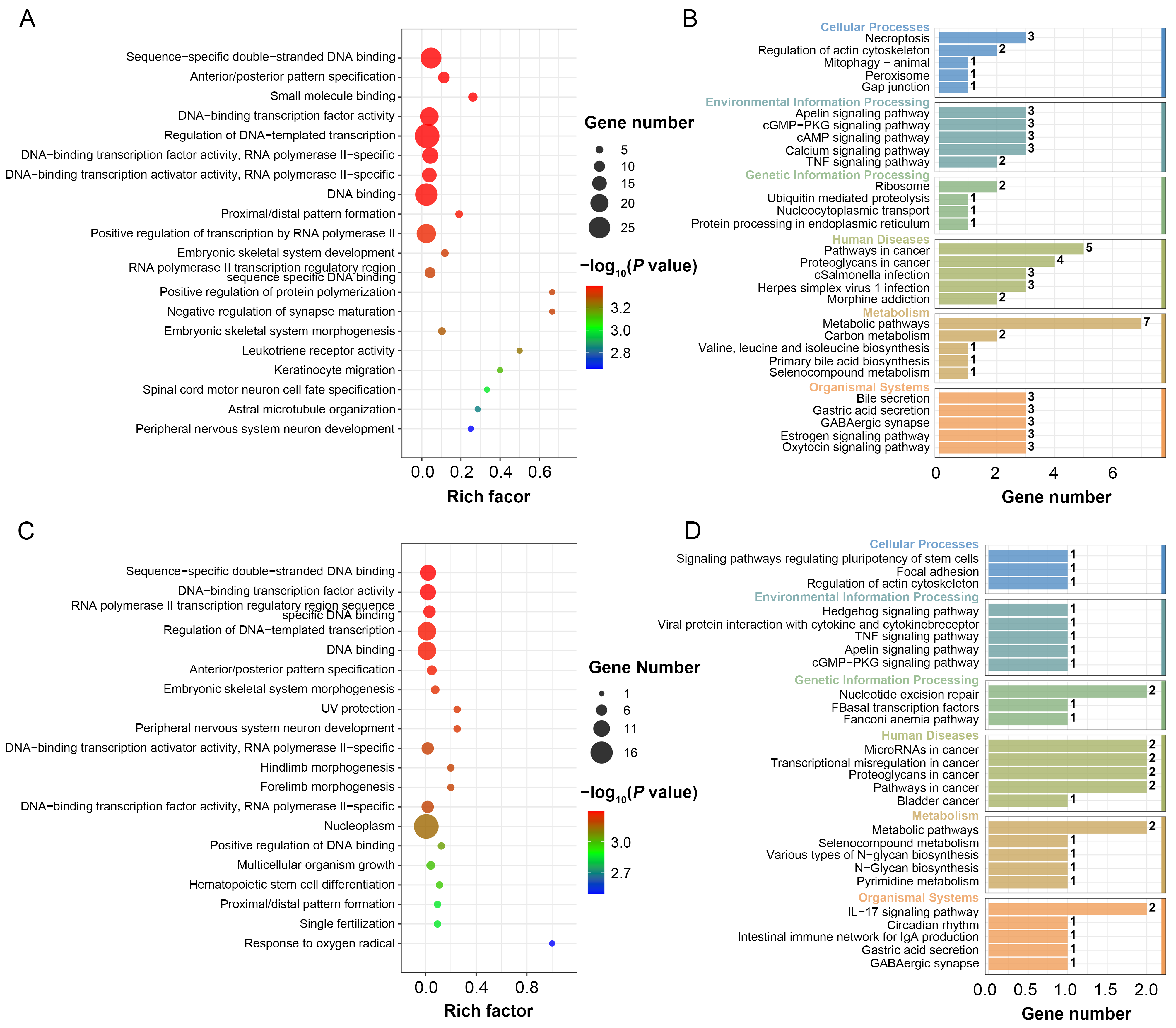

Supplement: Supplementary file 5 — Additional file 5: Fig. S1. GO and KEGG enrichment analyses of lncRNAs with two or more m6A peaks. A GO enrichment analyses of lncRNAs with two m6A peaks. B KEGG enrichment analyses of lncRNAs with two m6A peaks. C GO enrichment analyses of lncRNAs with 3 + m6A peaks. D KEGG enrichment analyses of lncRNAs with 3 + m6A peaks. [file 40104_2025_1164_MOESM5_ESM.tif]

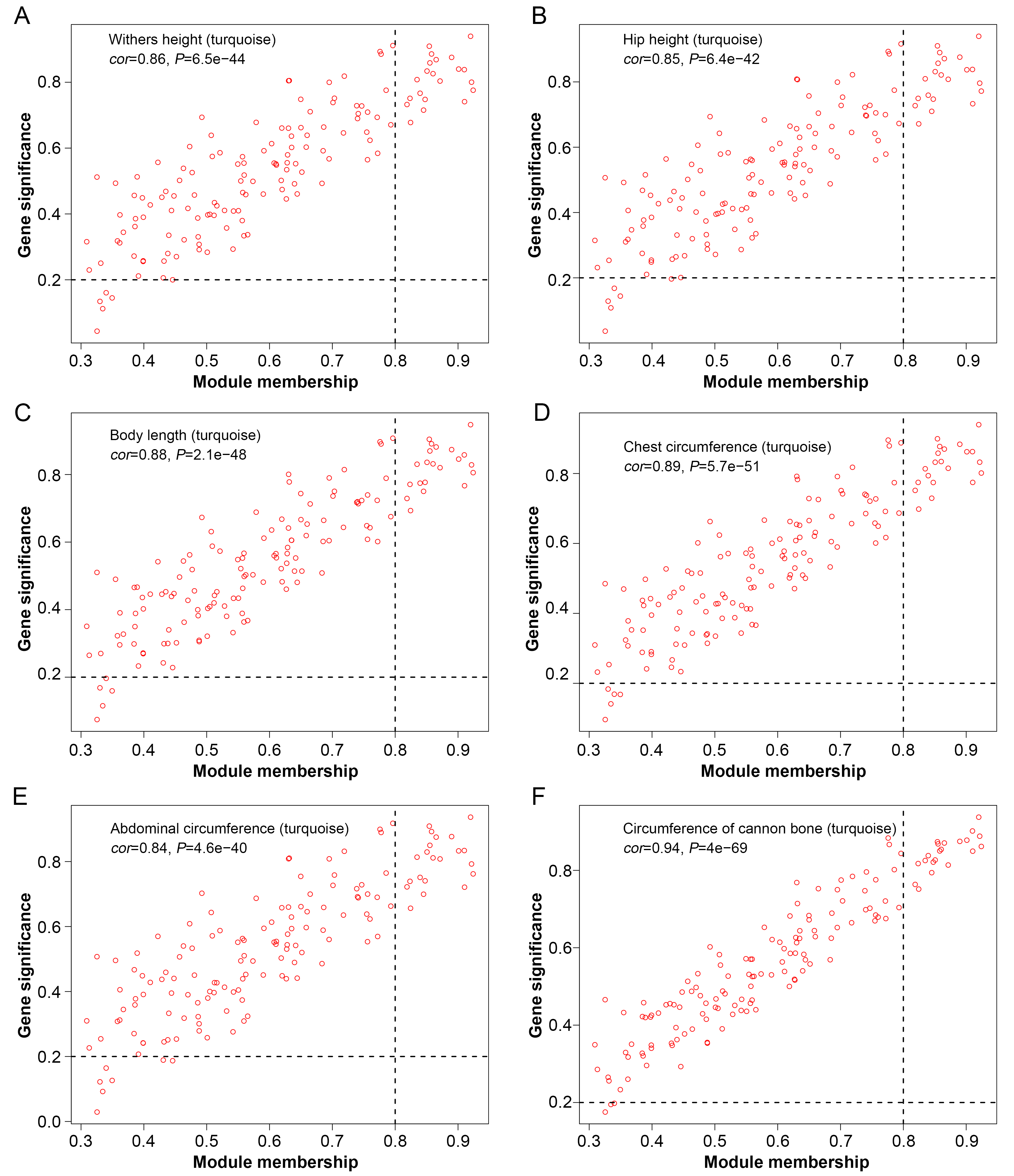

Supplement: Supplementary file 6 — Additional file 6: Fig. S2. Scatter plots of module eigengenes. A–F The genes of the turquoise module in the upper right were chosen as hub lncRNAs associated with withers height (A), hip height (B), body length (C), chest circumference (D) abdominal circumference (E) and cannon bone circumference (F) (module membership = 0.8, gene significance = 0.2). [file 40104_2025_1164_MOESM6_ESM.tif]
